# Advances in Focused Ultrasound for the Treatment of Brain Tumors

**DOI:** 10.3390/tomography9030090

**Published:** 2023-05-29

**Authors:** Rohan Rao, Anjali Patel, Kunal Hanchate, Eric Robinson, Aniela Edwards, Sanjit Shah, Dominique Higgins, Kevin J. Haworth, Brandon Lucke-Wold, Daniel Pomeranz Krummel, Soma Sengupta

**Affiliations:** 1 Department of Neurology and Rehabilitation Medicine, University of Cincinnati College of Medicine, Cincinnati, OH 45219, USA; 2 Department of Neurosurgery, University of Florida, Gainesville, FL 32608, USA; 3 Department of Neurosurgery, University of Cincinnati College of Medicine, Cincinnati, OH 45267, USA; 4 Department of Neurosurgery, University of North Carolina, Chapel Hill, NC 27599, USA; higginsd@email.unc.edu; 5 Department of Internal Medicine, Division of Cardiovascular Health and Disease, University of Cincinnati, Cincinnati, OH 45267, USA; 6 Department of Pediatrics, University of Cincinnati, Cincinnati, OH 45229, USA; 7 Department of Biomedical Engineering, University of Cincinnati, Cincinnati, OH 45219, USA

**Keywords:** microbubble-enhanced focused ultrasound, blood–brain barrier, blood–tumor barrier, immunotherapy, chemotherapy, glioma

## Abstract

Employing the full arsenal of therapeutics to treat brain tumors is limited by the relative impermeability of the blood–brain and blood–tumor barriers. In physiologic states, the blood–brain barrier serves a protective role by passively and actively excluding neurotoxic compounds; however, this functionality limits the penetrance of therapeutics into the tumor microenvironment. Focused ultrasound technology provides a method for overcoming the blood–brain and blood–tumor barriers through ultrasound frequency to transiently permeabilize or disrupt these barriers. Concomitant delivery of therapeutics has allowed for previously impermeable agents to reach the tumor microenvironment. This review details the advances in focused ultrasound in both preclinical models and clinical studies, with a focus on its safety profile. We then turn towards future directions in focused ultrasound-mediated therapies for brain tumors.

## 1. Introduction

Ultrasound has been a vital imaging modality since the 1970s [1]. Although ultrasound has primarily been used for rapid and cost-effective visualization of intra-abdominal, pelvic, and cardiac anatomy, and sparingly used in neurology and neurosurgery (i.e., transcranial and carotid Doppler) [2]. Its ability to transmit focused energy into soft tissues and utilize scattered energy to create pre- and post-operating images for interventional care has enhanced patient care and outcomes [3,4]. For example, ultrasound can assist surgical guidance in the operating room to allow the examination of various neurological pathways and diseases. Image formation relies on the transmission of ultrasound propagating through tissue at a rate of approximately 1.5 mm/µs, from which the penetration depth and outline of brain tumors can be calculated [4].

Aside from imaging, focus ultrasound (FUS) can be used to ablate tissue. FUS has regulatory approval with approved insurance reimbursement for neurologic diseases, such as benign essential tremor (ET) and Parkinson’s Disease (PD) [5,6,7]. For ET, Iorio-Morin et al. treated 10 patients with a unilateral FUS thalamotomy. Their results indicated that a majority of these patients experienced an improvement in their tremors with mild adverse effects of dysphagia following FUS treatment [5]. Similarly, Elias et al. reported hand-tremor improved after FUS thalamotomy with the most common adverse event being gait disturbance [7,8]. Mitigating these possible adverse effects could prove vital in the progression and increased use of this therapy in patients with benign ET. The benefit of thalamotomy for medication-refractory ET lasts up to 3 years with no progressive or delayed adverse effects [9]. Due to the progress of these clinical trials, the American Society of Stereotactic and Functional Neurosurgery (ASSFN) has published a set of best-practice statements to guide use of MR-guided FUS in treatment-refractory ET [10]. 

Similarly, for patients diagnosed with PD, randomized trials have shown that the use of FUS-mediated subthalamotomy can improve dyskinesias [6,11,12]. In patients for whom deep brain stimulation may be contraindicated, this serves as a less invasive alternative treatment route [13,14]. 

**Figure 1:**
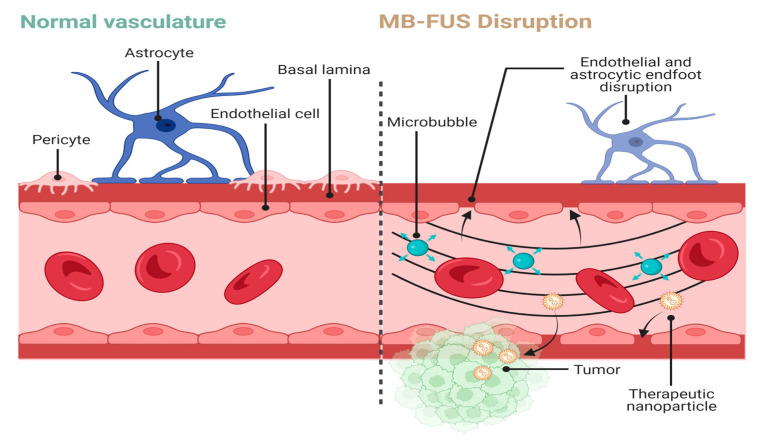
MB-FUS-mediated BBB disruption for therapy delivery. Normal vasculature consists of astrocytic endfeet surrounding endothelial cells linked by junctional complexes. MB-FUS disrupts these interactions to allow for passage of therapeutics into the tumor microenvironment. Arrows represent areas of disruption in the BBB. Created with BioRender.com.

In addition to directly being used as an ablative therapy, when combined with microbubbles, FUS can be used to transiently disrupt the blood–brain barrier (BBB) and brain–tumor barrier (BTB) and potentially provide patients an enhanced administration of treatments (Figure 1) [15,16,17]. Microbubbles are nano to micron sized, gas-filled particles that volumetrically oscillate when exposed to ultrasound. The gas is commonly encapsulated in a lipid shell, though protein and polymer shells have also been explored [18]. The anatomy of a healthy BBB involves many cells that regulate the transport of osmotically active molecules between the brain parenchyma and surrounding vasculature [19]. Brain capillaries are surrounded by pericytes, microglia, astrocytes, and junctional complexes (tight, adherens, gap) that mediate permeability, monitor immune cell infiltration, and regulate flow of cerebrospinal fluid (CSF) [20]. Selective transmembrane proteins, such as P-glycoproteins, and efflux transporters can use ATP to actively export intraparenchymal solutes. In contrast, the BTB is created by primary or metastatic cancer cells initiating angiogenesis to form a unique vascular network directly surrounding the tumor. Much of this neurovasculature is leaky as tumor angiogenesis fails to faithfully recreate BBB features, such as tight junctions [21]. Furthermore, Sprowls et al. reported that tumors can disrupt the BBB through down-regulation of junction proteins such as Mfsd2a [19]. Tumors also induce a pro-inflammatory state which promotes microglial secretion of VEGF, inducing a vasogenic edema [19,22]. These studies highlight the complex interplay between tumor tissue and its vascular supply which can confound the effective delivery of therapeutics. 

The BBB and BTB are clinically important as they limit the passage of therapeutics into the tumor microenvironment (TME) [23,24,25,26]. For example, Ranjan et al. detailed stem cell assay-guided chemotherapy for patient-specific glioma subtypes, but these chemotherapeutic regimens are limited by the impermeability of the BBB [27]. Significant advances have been made in the preclinical delivery of glioma therapeutics using focused ultrasound (FUS). Currently, the DNA alkylator, Temozolomide (TMZ), is one of the few chemotherapeutics which has the capability of bypassing the BBB because of its small size and lipophilicity [28]. It is now the gold standard for adjuvant treatment in several brain tumors [29]. However, even with its ability to bypass the BBB, effective delivery of TMZ to the TME is further impeded by the heterogenous blood supply to the tumor bulk. In particular, the increased interstitial fluid pressure (IFP) between the tumor cells and their blood supply leads to heterogenous concentrations of chemotherapy within the tumor bulk [30,31]. With these barriers and the consequent poor bioavailability of TMZ in the TME, TMZ must be delivered more frequently, increasing the risk of adverse side effects. However, microbubble-enhanced focused ultrasound (MB-FUS) has promise to solve this bioavailability issue as molecules, such as doxorubicin and trastuzumab, have been reported to have increased uptake patterns when MB-FUS was utilized to disrupt the BBB/BTB barriers [19,32,33]. 

This review will further expand upon the advances in using MB-FUS as a preclinical and clinical method in the disruption of the BBB/BTB for the treatment of brain tumors while analyzing the safety measures that need to be taken into consideration [4]. 

## 2. Mechanisms behind MB-FUS-Mediated BBB Disruption and Permeabilization

FUS is a powerful tool for modifying the BBB and BTB. FUS traditionally has two modes, high-intensity and low-intensity. High-intensity FUS can quickly raise the temperature of tissues allowing for precise and targeted thermal ablation [34]. Coagulative necrosis is often the result of thermal ablation [35]. Low-intensity FUS, in combination with microbubbles, can safely disrupt the BBB in a manner that does not cause significant irreversible damage to peripheral tissues and cells [34]. 

### 2.1. Microbubble Composition and Characteristics

Low intensity FUS-mediated disruption of the BBB requires exogenous microbubble administration for safe, transient permeabilization. Exogenous microbubbles are comprised of gas encapsulated by protein, lipids, or polymers with commercially available microbubbles ranging from 1 to 10 m [36,37]. Their composition can vary greatly in ratios of polymers, proteins, lipids, and gases [34,35]. They are commonly administered as ultrasound contrast agents [35].

Microbubbles are an essential component of FUS-mediated BBB disruption. They convert ultrasound (US) energy, effectively reducing the amount of required US energy to induce BBB disruption compared to FUS alone [38]. Microbubbles are highly compressible and cavitate under ultrasound [39]. Cavitation is the phenomena whereby microbubbles oscillate and collapse due to harmonics [35].

Two types of cavitation exist, inertial and stable. Inertial cavitation is often associated with higher ultrasound intensities. At these intensities, microbubbles expand and collapse, with the collapse being dominated by the inertia of the surrounding fluid [34]. When they collapse, they can produce jetting, free radicals, and shock waves [34,40]. Consequently, nearby blood vessels can be damaged resulting in petechiae, ischemia, or hemorrhage [34]. Stable cavitation typically occurs when microbubbles oscillate with smaller amplitudes within the ultrasound field. These oscillations mediate the transfer of energy to the local fluid environment through microstreaming around the bubble and shear stress imparted on nearby vessel walls [34,40]. Stable cavitation, induced by low and high frequencies, is the driver of contemporary BBB disruption investigations [35]. It can temporarily disrupt the junctional complexes of the endothelial cells within the BBB for up to 24 h depending on the parameters used [41,42,43]. One means by which this occurs is the deformation of cellular membranes and subsequent activation of mechanosensitive ion channels which increase membrane permeability (Table 1) [41].

### 2.2. Post Low-Intensity FUS Cellular and Biochemical Changes

Low-intensity FUS stimulates a variety of intracellular biochemical responses apart from the physical effects of cavitation. These include, but are not limited to, reductions in gap and tight junction proteins [35], potentiation of transcytosis [29], and downregulation of P-glycoprotein [36]. FUS is also hypothesized to induce changes to BBB permeabilization regulatory pathways, such as the phosphatidylinositol 3-kinase/Akt pathway [35]. Cellular adhesion molecules (CAMs) are another cellular component of the BBB which have been shown to aid in the transmigration of CD4+ T-cells across the BBB via an interaction with T-cell associated lymphocyte function-associated antigen 1 (LFA-1) [44]. Interestingly, FUS upregulates intercellular adhesion molecule 1 (ICAM1) for 24 h [45]. This transient upregulation may play a role in the FUS-mediated immunostimulation discussed in later sections. While many of the biophysical effects of FUS have been studied, there remains some gaps in knowledge. For example, the effects of FUS on adherens junctions, a key component of the BBB, remains poorly studied [46].

## 3. MB-FUS as a Delivery Method (Preclinical)

The first step in applying the technology in a rodent model is to have proof of concept of MB-FUS safety and efficacy. This experimentation involves the delivery of intravascular optical dyes, such as Evans blue or FITC-Dextran, followed by MB-FUS permeabilization. This allows for direct visualization of dye extravasation into the tumor parenchyma. Englander et al. used a primary glioma murine model to deliver Evan’s blue dye via MB-FUS permeabilization [47]. It is important to note the differing MB-FUS parameters used in each study because delivering higher intensity US energy has the potential to be neurotoxic. Contrastingly, lower intensity US may not effectively permeabilize the BBB. Magnetic resonance imaging (MRI) contrast extravasation into the parenchyma was confirmed via MRI on day 14 post-injection and sonication. Importantly, the researchers found no impairment in motor or cardiopulmonary function in the MB-FUS-treated group compared to control mice, supporting the safety of MB-FUS [47,48]. With success in murine models, researchers have transitioned to primate models to test FUS [49,50]. Extending MB-FUS methodology between species is difficult due to the differences in anatomy (i.e., skull thickness) and physiology [51]. Despite these obstacles, several groups have proven the efficacy and safety of delivering MRI contrast via MB-FUS in primates [51,52,53]. Advances have also been made in a porcine model using MB-FUS with a closed-loop feedback controller to induce BBB opening while avoiding hemorrhage [54].

Objectives have changed from delivering innocuous payloads, such as dyes, to delivering therapeutic-containing payloads. The following subsections discuss preclinical models for MB-FUS delivery of the three main classes of tumor therapeutics—chemotherapy, immunotherapy, and RNA-based therapeutics (Table 2). 

### 3.1. Chemotherapy Delivery via MB-FUS

As mentioned previously, chemotherapy has notorious difficulty in permeating the BBB. Lipinski et al. was one of the first groups to identify a set of rules which can predict a molecule’s ability to penetrate the BBB [66]. They predict poor penetration when there are more than 5 H-bond donors, 10 H-bond acceptors, or the molecular weight is greater than 500 Da. TMZ is one of the few chemotherapeutics which satisfies these conditions [67]. Given the limited arsenal of chemotherapeutics for brain tumors, MB-FUS offers a promising avenue to expand the use of FDA-approved chemotherapeutics into the brain. 

Etoposide is a chemotherapeutic which functions by inhibiting the binding of topoisomerase II to DNA, leading to DNA breaks [68]. Outside of TMZ, etoposide has one of the higher BBB penetrations among other chemotherapeutics, making it a leading candidate for delivery via MB-FUS [69]. MB-FUS-mediated etoposide delivery in a murine model led to 45% reduction in tumor growth and eight-fold increase in etoposide brain tumor concentration compared to delivery without MB-FUS [47,55]. Similarly, nanoparticles carrying cisplatin delivered via MB-FUS demonstrated a 28-fold increase in intratumor chemotherapeutic concentration and survival benefit when compared to non-FUS control [56]. Even with the already-BBB permeable TMZ, MB-FUS delivery increases the CSF:plasma ratio of TMZ by 16% and marginally extends median survival [57,58]. 

However, not all FUS-delivered chemotherapy has had positive impacts on survival in murine models. For example, irinotecan, another topoisomerase inhibitor, delivered by MR-guided FUS did not increase survival in an F98 glioma model [59]. The delivery was shown to be safe despite not having a survival impact. Of note, this study did not utilize microbubbles, and the researchers comment that the FUS parameters need to be optimized for future studies. In this same rat model, McDonnald et al. was able to successfully deliver carboplatin with a statistically significant increase in overall survival [60]. This group used MB-FUS, which the irinotecan study did not, highlighting the necessity of tight parameter control for successful cavitation. 

### 3.2. Immunotherapy Delivery via MB-FUS

While chemotherapy has been historically the most common cancer therapy, it is a nonspecific treatment which leads to significant systemic toxicity. Immunotherapy serves as a highly specific therapy in which the body’s immune system is trained to recognize and attack tumor antigens. Challenges such as the lack of tumor-specific antigens and the immune-privileged nature of the brain parenchyma have slowed advances in brain tumor immunotherapy [70,71]. However, the discovery of lymphatic vessels in the meninges and the increasing evidence of T lymphocyte penetration of the BBB have reinvigorated research in immunomodulation for the treatment of brain tumors [72,73]. The same obstacles to delivery as faced by chemotherapeutics still exist for immunotherapy with MB-FUS serving as a potential solution. 

Tumor cells often express programmed cell death ligand-1 (PD-L1) which binds to its receptor (PD-1) on T-cells. This interaction leads to tumor immune escape. As such, it has been shown that high levels of PD-L1 in glioblastoma are correlated with poor survival in patients [74,75]. The use of antibodies against PD-L1/PD-1 has been shown to be effective in reducing tumor burden in a number of extracranial solid cell tumors [76,77]. MB-FUS was shown to enhance the penetrance of anti-PD-1 in GL261 mouse gliomas and improve survival [61,62]. Interestingly, both the studies involving anti-PD-1 implemented a tumor challenge at later tumor survival stages in which tumor cells were redelivered into mice along with anti-PD-1 and MB-FUS therapy. Both groups found a significant survival benefit in tumor rechallenged mice which suggests there is a lasting memory T-cell response following initial anti-PD-1 delivery. 

Interestingly, MB-FUS sonication of the BBB leads to a immunostimulatory effect through increased dendritic and lymphocytic penetration of the TME [78,79]. This effect is not novel to FUS-treated brain tumors. Other groups have shown that various immune cell subpopulations become upregulated in the tumor microenvironment following FUS treatment [80]. In pancreatic cancer, the FUS-mediated immunostimulation enhanced cell-mediated immunity [81]. The consequence of this immunostimulation in glioma requires further study regarding the direct impact on tumor growth in both preclinical and clinical models. Additionally, there could be a synergistic effect between FUS-mediated immunostimulation and the delivery of exogenous immunotherapy. 

### 3.3. RNA-Based Therapeutic Delivery via MB-FUS

With the advent of mRNA vaccines for COVID-19 and popularity of CRISPR-Cas9 technology, RNA-based therapeutics have gained significant traction as a therapeutic modality. The advantage RNA therapeutics have over chemotherapy or immunotherapy is that they are much smaller, allowing for greater penetrance with MB-FUS. However, the detriment of RNA therapies is that they are highly unstable in peripheral circulation due to degradation by endogenous RNAases [82,83]. This instability requires packaging in a lipid nanoparticle which reintroduces the issue of poor BBB penetration. Given the novelty of RNA therapeutics, there have been fewer clinical or pre-clinical trials involving MB-FUS delivery of RNA-based therapy compared to chemo- or immunotherapy. 

RNA interference (RNAi) is an endogenous, real-time RNA editing tool. The three subclasses of RNAi include small interfering RNA (siRNA), short hairpin RNA (shRNA), and micro RNA (miRNA) which can be exogenously delivered to silence targeted mRNA sequences [84,85,86]. In a medulloblastoma mouse model, Guo et al. employed MB-FUS to aid delivery of an siRNA targeting the mRNA coding for the Smoothened (SMO) protein, whose function is critical to the Sonic Hedgehog Pathway [64]. This study was particularly impactful as they provided details on optimizing lipid nanoparticle formulation for effective RNA packaging. Most importantly, the payload delivered with the help of MB-FUS led to increased medulloblastoma apoptosis when compared to siRNA nanoparticles delivered without MB-FUS [64].

Clustered regularly interspaced short palindromic repeat (CRISPR)-associated protein 9 (CRISPR/Cas9) can generate double-stranded breaks in target DNA with subsequent insertion or deletion of desired sequences [40]. One gene relevant to glioma treatment is *O6-methylguanine-DNA methyltransferase* (*MGMT*). The MGMT protein reverses the DNA damage induced by TMZ [41,42,43]. Therefore, methylation or inactivation of *MGMT* confers greater sensitivity to TMZ. Yang et al. delivered a CRISPR/Cas9 plasmid encapsulated in a lipid nanoparticle via MB-FUS to target *MGMT* in a mouse model of glioblastoma [65]. Mice treated with this regimen had increased sensitivity to TMZ and prolonged survival compared to TMZ treatment alone. 

## 4. MB-FUS as a Delivery Method (Clinical)

The continuous non-fenestrated capillaries of the BBB prevents the entry of neurotoxic molecules [87]. Although this is advantageous in reducing pathogens and preserving homeostasis, it is a barrier to the delivery of pharmaceutical treatments [88]. With each successive administration of MB-FUS, the force needed to disrupt the tight junctions of the capillaries reduces. This way, the BBB is more efficiently opened for the improved uptake of drugs to the brain. Various systems exist which serve to deliver MB-FUS to disrupt the BBB and improve the delivery of drugs [89].

### 4.1. Focused Ultrasound Commercial Systems

**Figure 2:**
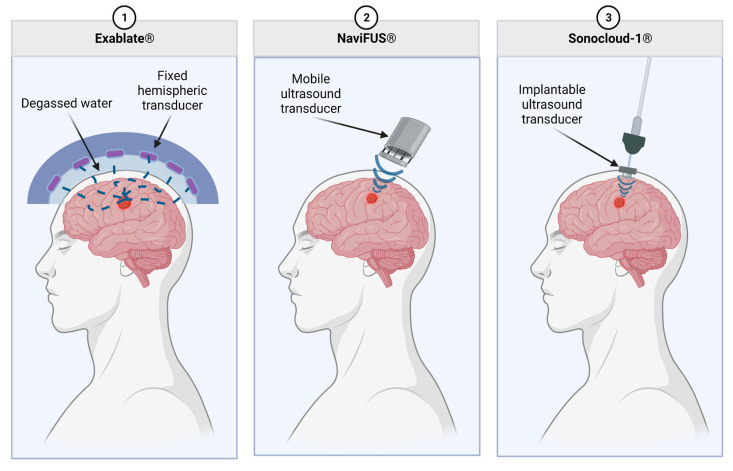
Commercially available MB-FUS modalities. Exablate uses a fixed, hemispheric transducer which is separated from the patient by a layer of degassed water. The NaviFUS is a portable ultrasound transducer which allows for slightly more flexibility in treating various tumor localizations. Lastly, the Sonocloud-1 has an implantable transducer which connects to external equipment. This allows for a highly reproducible dose to be delivered to the TME over multiple treatments.

There are different systems of delivering FUS to increase the permeability of the BBB, including Exablate Neuro, Sonocloud-1/9, and NaviFUS (Figure 2). As described by Chen et al., Sonocloud-1 by CarThera requires a transcranial implant for the delivery of ultrasound to the BBB. While implanted, this device delivers a 25,000-cycle pulse at 1 Hz for 4 min. CarThera has also created an upgraded Sonocloud-9 system which uses nine transcranial implants for the ability to deliver more localized US intensity. With Exablate, a hemi-spherical transducer for the delivery of the ultrasound is placed over the top of the skull with 1024 transducer elements delivering a frequency of 620–720 kHz [90]. The Exablate requires the concurrent use of MRI with FUS for imaging [79]. MRI imaging is used to correctly localize the region to target for therapy. Microbubbles are also delivered intravenously for the improved permeability of the BBB [91]. NaviFUS allows personalized modulation of ultrasound intensity and amplitude for the individual patient. It also contains neuronavigation that helps target ultrasound to the tumor [79]. 

These various methods each have their advantages and disadvantages. NaviFUS benefits from less invasiveness, user-friendliness, and minimal extra equipment, such as an MRI [88]. Sonocloud-1/9 has the benefit of ease of repeated treatments. Exablate theoretically has more control over the intensity and localization of FUS given the increased number of transducers and MRI guidance.

### 4.2. Chemotherapy Delivery via MB-FUS

Idbaih et al. used MB-FUS to deliver IV carboplatin to improve drug delivery for glioblastoma patients. This was accomplished with the SonoCloud-1 system where the transducer was placed via a burr hole through the skull bone overlying the tumor area at the external face of the dura mater [92,93]. As this was an escalating ultrasound experiment, the ultrasound pressure was gradually escalated from 0.41 MPa to 1.15 MPa at seven different levels [92]. The investigators of this experiment ensured at least three subjects were treated with each of the seven levels of the ultrasound pressure [92]. Patients recruited had recurrent de novo GBM after being treated with standard of care (radiation with adjuvant TMZ). Patients received IV 0.1 mL/kg SonoVue Microbubbles followed by pulsed ultrasound frequency of either 0.5 or 1 Hz through the SonoCloud-1 implant for 150–270 s. Immediately following MB-FUS, patients received an IV carboplatin (AUC4, AUC5, or AUC6) infusion for 60–90 min. Confirmation of BBB opening was done via scheduled post-treatment MRI. This treatment regimen occurred every four weeks for a maximum of six treatments [92]. While carboplatin is used as a third-line treatment for GBM, it has been shown to reduce tumor size in glioblastoma [92,94,95,96,97,98,99,100,101]. Therefore, by increasing the permeability of the BBB through MB-FUS, they hypothesized intratumoral carboplatin concentration would increase [92].

For those who obtained at least a grade 2 opening of the BBB as quantified by post-treatment MRI, the progression free survival (PFS) was 4.11 months, and the overall survival (OS) was 12.94 months [92]. Among patients with insufficient BBB permeabilization, PFS was 2.74 months, and OS was 8.64 months [92]. This demonstrates a correlation between achieving an increased opening in the BBB with concomitant chemotherapy delivery and improved survival.

During this experiment, 67% of the adverse effects were graded as 1 or 2 according to the CTAE. The most common adverse effects were hematological disorders at 32% and fatigue at 19%. Dose limiting toxicities were not apparent during or after the course of the treatments. Among central nervous system (CNS) adverse effects, the most common were headaches (26%), cerebral edema (11%), and syncope (11%). Few patients presented with transient facial palsy which improved within two hours after corticosteroid treatment. The most severe adverse effect observed was grade 4 edemas in two patients (11%). In both cases, symptoms resolved within two hours after corticosteroid therapy. After weighing the risks and benefits of MB-FUS-mediated carboplatin delivery, there appears to be a therapeutic benefit. Further clinical trials will need to be conducted with other chemotherapeutics and varying MB-FUS settings. 

Another phase I trial used the updated Sonocloud-9 system to deliver albumin-bound paclitaxel in patients with recurrent GBM [102]. Patients underwent MB-FUS every three weeks for up to six cycles with dose escalation of paclitaxel up to 260 mg/m^2^. Given that this was a phase I trial, the researchers were only able to comment on safety. The main severe adverse effect noted was self-resolving encephalopathy in one patient with several patients experiencing mild headache as the predominant side effect. A phase II trial is ongoing. 

### 4.3. Immunotherapy Delivery via MB-FUS

Current trials are underway to examine the efficacy of pembrolizumab (Keytruda) with the use of Exablate for metastatic brain cancer. Pembrolizumab is shown on its own to improve clinical outcomes in patients with metastatic glioblastoma [103]. The treatment will be provided every 3 weeks with the use of Exablate preceding the infusion of pembrolizumab to target the BBB for improved uptake [104]. The primary outcome is a response of tumor burden compared to baseline as measured by MRI every three weeks for a total of six months. 

### 4.4. Ongoing Trials

The Toronto group is focusing on ultrasound induced capability to obtain liquid biopsies in the BRAINFUL Trial [105]. From a treatment standpoint, several groups are looking into treatment for Parkinson’s disease, movement disorders, temporal lobe epilepsy, and neurodegenerative dementias. Exablate is also being investigated for diffuse intrinsic pontine gliomas and brain metastasis at high frequency with and without chemotherapy regimens. Initial safety studies have been demonstrated for glioma and FUS-mediated chemotherapy delivery [106,107]. Emerging innovation is being investigated in terms of anxiety, depression, and pain relief. At low intensity, focused ultrasound is being utilized for memory enhancement and stroke rehabilitation. From a more mechanistic standpoint, several groups are looking at BBB disruption and association with glymphatic clearance [108].

## 5. Safety of MB-FUS

The use of MB-FUS to disrupt tight junctions between endothelial cells in the BBB and BTB results in a transient inflammatory response due to microbubble induced cavitation and pulsed thermal damage [109,110]. Safety concerns for the use of MB-FUS include issues related to this inflammatory response and subsequent edema. 

### 5.1. Inflammation

It is hypothesized that stress exerted on microvascular walls by oscillating microbubbles at the focus of the ultrasound beam, as well as thermal damages incurred by the acoustic pulse initiate an acute, transient inflammatory response in the vascular endothelium [111]. This response leads to an increase in proinflammatory cytokine and chemokine gene expression, contributing to many of the reported effects of MB-FUS, including neurogenesis, angiogenesis, altered transporter expression, increased endocytosis, and reduced immunoreactivity of tight junction proteins. The extent of neuroinflammation is dependent on ultrasound intensity, pulse repetition frequency, and sonication which can clinically manifest as intracerebral hemorrhage, transient edema, necrosis, and reactive gliosis [112]. The degree of inflammatory response appears to be independent of MB concentration [113].

The brain microvessels experience the largest magnitude of stress during sonication. However, different types of cells have varied responses to sonication. Activation of vascular endothelial cells results in release of proinflammatory cytokines and chemokines, promoting infiltration of leukocytes across the BBB. Acute activation of astrocytes may play a role in neuroprotection and homeostasis during acute ischemia. Pouliopoulos et al. showed that the use of MB-FUS to bypass the BBB triggers a short-lived immune response in the targeted region with increased microglia density on day 2 that resolved by day 18, without clinically measurable deficits, in a non-human primate model [114]. They also noted enhanced immature neuron presence in areas that underwent BBB opening, compared to areas with intact BBB [114]. Others have shown microstructural changes identified one year after lesion, representing gross tissue reorganization [115].

When stimulated by physical stress, the CNS vascular endothelium produces pro-inflammatory cytokines and chemokines to protect the brain from further damage. After induction of thermal damage due to use of MB-FUS there is evidence of the transcription of pro-inflammatory cytokines, returning to baseline after 24 h [111]. This may explain the usefulness of MB-FUS for driving transient angiogenic processes and reducing drug efflux. Sterile inflammatory response in the parenchyma, as indicated by damage-associated molecular pattern (DAMP) response and elevated HSP, IL-1, IL-18, and TNF alpha, lasts approximately 24 h, with macrophages being detected six days after treatment [45].

Secondary damage can occur when inflammation becomes chronic. The pro-inflammatory response dampens and returns to baseline after 24 h, which may be regulated by astrocyte-derived immunoregulatory cytokines and astrocyte-microglial crosstalk. Transiently controlled levels of inflammation can promote myelin debris clearance, myelin repair, angiogenesis, and amyloid beta plaque clearance. When inflammation becomes chronic, it suppresses neurogenesis and leads to apoptosis, necrosis, and other neurodegenerative processes. Researchers have characterized safety profiles to optimize efficacy of BBB disruption, while minimizing chronic inflammation and its detrimental sequelae [116,117].

### 5.2. Edema

MB-FUS exerts tissue damage to a localized area of focus through three concentric zones, a hypodense center of coagulative necrosis, a surrounding hyperintense region of cytotoxic edema, and a weakly hyperintense periphery of vasogenic edema [118,119]. As mentioned previously, using larger microbubbles or increasing MB-FUS intensity will lead to a longer BBB permeabilization time at the risk of increased edema. Lesion size is dependent on accumulated thermal dose [110,120]. In a rabbit model, cerebral edema developed and culminated over 48 h, diminishing over five days [121]. Histologic analysis showed central necrosis in white matter surrounded by edematous tissue with inflammatory cells [122]. In a study of 21 patients with glioblastoma treated by implanted FUS sonographic device for drug delivery, two (11%) experienced steroid-responsive edema that resolved within several hours [92]. While there may be some concern for edema after the use of MB-FUS, it is generally considered to have a good safety profile. In fact, low-intensity US stimulation has been shown to attenuate BBB disruption and decreased edema in a mouse model of ischemic stroke [123].

## 6. Conclusions and Future Directions

The ability of MB-FUS to destabilize the BBB and facilitate delivery of antitumor agents represents a promising avenue in glioma treatment. In preclinical models, MB-FUS has been shown to safely and transiently permeabilize the BBB for the delivery of diverse payloads, including traditional chemotherapy, immunotherapy, and RNA-based therapeutics. In clinical studies, MB-FUS has also shown promise and there are a significant number of trials underway. Future preclinical and clinical research directions should explore further potential detrimental effects of MB-FUS, such as brain inflammation and uncontrolled edema, and employing approaches to prevent them from occurring. In addition, improving the invasiveness of various commercial systems of MB-FUS, such as Exablate, Sonocloud-9, and NaviFUS, could improve patient satisfaction and outcomes. Several clinical trials are in the recruitment phase to further test the efficacy of these technologies in humans, as highlighted above. To improve efficacy, focused chemotherapy delivery through endovascular microcatheters should be investigated. Drugs can be delivered directly to the target region of disruption through microcatheter selective administration. Understanding the role of MB-FUS in tumors of neurological origin can aid in decreasing comorbidities while improving clinical outcomes. 

## Figures and Tables

**Table 1:** Post-low intensity FUS changes.

	Post Low-Intensity FUS Changes
Structural	Deformation of cellular membrane
	Disruption of junctional complexes
	Potentiation of transcytosis Reduction in junctional complexes
Cellular and Biochemical	Downregulation of P-glycoprotein
	Altered BBB permeability regulatory phosphatidylinositol 3-kinase/Akt pathway Upregulation of cellular adhesion molecules

**Table 2:** MB-FUS-mediated therapy delivery in preclinical models. Most research has focused on classic chemotherapy, but more recent advances have occurred in delivery of immunotherapy and RNA-based therapeutics.

Therapy	Animal Model	Outcome	Reference
Etoposide	Mouse MGPP3 GBM	MB-FUS increased etoposide concentration in brain tumor tissue by eight-fold with subsequent 30% increase in MOS.	[55]
Cisplatin	Mouse F98 glioma	MB-FUS increased penetrance of nanoparticles loaded with cisplatin in a mouse model and improved survival compared to cisplatin nanoparticles without MB-FUS.	[56]
Temozolomide	Rat 9L gliosarcoma	MB-FUS delivery increases the CSF:plasma ratio of TMZ by 16% and marginally extends median survival.	[57,58]
Doxorubicin	Rat 9L gliosarcoma	24% greater median survival time in rats treated with MB-FUS and doxorubicin compared to nontreated rats (*p* = 0.0007)	[33]
Irinotecan	Rat F98 glioma	FUS-delivered irinotecan did not improve overall survival but was safely delivered. This study did not use microbubbles to aid FUS.	[59]
Carboplatin	Rat F98 glioma	Tissue-to-plasma ratios of carboplatin was increased by 2.9 times after MB-FUS.	[60]
Anti-PD-1/Nivolumab	Mouse GL261 glioma	MB-FUS enhances the delivery of anti-PD-1 and improves overall survival.	[61]
Anti-PD-1/Nivolumab	Mouse GL261 glioma	MB-FUS increased survival in anti-PD-1 mice compared to control. Interestingly, FUS-mediated anti-PD-1 therapy was more effective when performed at a later timepoint when tumors were well-established.	[62]
Anti-PD-L1/Pembrolizumab	Mouse GL261 glioma	MB-FUS enhanced intranasal delivery of anti-PD-L1 without testing the treatment efficacy in GL261 mice.	[63]
*SMO* siRNA	Mouse SmoA1-Math1-GFP	MB-FUS delivery of *SMO* siRNA led to increased medulloblastoma apoptosis as measured by TUNEL staining.	[64]
CRISPR/CAS9 targeting *MGMT*	Mouse T98G NOD-SCID	Mouse model of GBM was treated with CRISPR/Cas9 targeting MGMT to resensitize tumor cells to TMZ. CRISPR/Cas9 delivered by MB-FUS led to increased TMZ sensitivity and improved overall survival compared to TMZ alone.	[65]

## Data Availability

Not applicable.
